# MUC16 impacts tumor proliferation and migration through cytoplasmic translocation of P120-catenin in epithelial ovarian cancer cells: an original research

**DOI:** 10.1186/s12885-019-5371-4

**Published:** 2019-02-22

**Authors:** Xin Chen, Xiaoduan Li, Xinjing Wang, Qinyi Zhu, Xiaoli Wu, Xipeng Wang

**Affiliations:** Department of Gynecology and Obstetrics, Xin Hua Hospital, Shanghai Jiao Tong University School of Medicine, 1665, Kongjiang Road, Yangpu District, Shanghai, 200000 People’s Republic of China

**Keywords:** Epithelial ovarian cancer, MUC16, p120-catenin, Proliferation, Migration

## Abstract

**Background:**

Epithelial ovarian cancer (EOC) remains one of the most lethal gynecologic cancers, and its pathogenetic mechanism remains unclear. Here we show that MUC16 promotes the translocation of p120-catenin (p120ctn) to the cytoplasm and consequently activates ras homolog (Rho) GTPases RhoA/Cdc42 activation to modulate the proliferation and migration abilities of EOC cells.

**Methods:**

We collect 94 ovarian cancer (OC) patients’ tissue samples to constitute tissue microarray (TMA) and analyze the MUC16 and p120ctn expression levels. Lentivirus transfection is used to overexpress cytoplasmic tail domain (CTD) of MUC16 and CRISPR/Cas9 genome-editing system is firstly used to knock out MUC16 in EOC cells. The proliferation or migration ability of cells is analyzed by MTS or migration assay.

**Results:**

We find that MUC16 and p120ctn are aberrantly overexpressed in 94 clinical OC samples compared with benign ovarian tumors (BOT). MUC16 is a critical inducer of the proliferation and migration of EOC cells and the CTD of MUC16 plays an important role during this process. In addition, we reveal the relationship between MUC16 and p120ctn, which has not previously been studied. We show that MUC16 promotes the translocation of p120ctn to the cytoplasm and consequently activates Rho GTPases to modulate the proliferation and migration abilities of EOC cells. The cell proliferation and migration abilities induced by MUC16 are mediated by p120ctn through RhoA/Cdc42 activation.

**Conclusions:**

The highly expressed MUC16 promotes the translocation of p120ctn to the cytoplasm, where it activates RhoA/Cdc42 to modulate the proliferation and migration abilities of EOC cells. These findings may provide new targets for the treatment of EOC.

**Electronic supplementary material:**

The online version of this article (10.1186/s12885-019-5371-4) contains supplementary material, which is available to authorized users.

## Background

Mucin-16 (MUC16, CA125) is a type I transmembrane protein. It is the largest member of the mucin family, which comprises high-molecular-weight glycoproteins synthesized by human epithelial cells to protect and heal the epithelial surfaces [1]. MUC16 contains more than 22,000 amino acids and is composed of an extracellular N-terminal region, a heavily glycosylated region of tandem repeat domains interspersed with sea urchin sperm protein, enterokinase, and agrin (SEA); a transmembrane region; and a short cytoplasmic tail domain (CTD) of 32 amino acids [2]. The extracellular portion of MUC16 can be cleaved off and released into the serum, becoming the circulating marker CA125, which is a well-known marker for the recurrence of EOC. MUC16 is a useful marker not only for clinical diagnosis but also for prognosis: MUC16 overexpression on the surface of cancer cells is correlated with poor outcome in pancreatic, colon and EOC patients [3, 4]. MUC16 has a critical pro-tumorigenic role in EOC [5], especially the CTD of MUC16 [6, 7].

P120 catenin (p120ctn, also known as CTNND1) was originally discovered in 1989 as a 120 kDa substrate of the oncogenic Src tyrosine kinase [8]. P120ctn, δ-catenin (CTNND2), p0071 (PKP4), and ARVCF (armadillo repeat gene deleted in Velo-Cardio-Facial Syndrome) make up the subfamily of armadillo (ARM) repeat-containing proteins [9]. P120ctn is composed of four characteristic functional domains, namely, a short C-terminal tail, an ARM domain, a regulatory or phosphorylation domain and an N-terminal coiled-coil domain [10]. P120ctn is well known for associating with the juxta membrane domain of the cadherin cytoplasmic tail to suppress cadherin endocytosis and to regulate the actin cytoskeleton via the central ARM domain in mammals [10–12]. Apart from stabilizing cadherins in cell-cell adhesion at the plasma membrane, p120ctn can also translocate to the cytoplasm and nucleus to affect downstream signaling, thereby influencing cell proliferation, invasion, migration, inflammation and innate immunity [12]. Interestingly, p120ctn exerts not only pro-tumorigenic but also anti-tumorigenic functions in cancer. Loss of p120ctn in various epithelial tumors induces epithelial-mesenchymal transition (EMT), which turns cancer cells motile and invasive [13]. On the other hand, p120ctn’s function of maintaining cadherin-mediated cell-cell junctions prevents suspended cancer cells from undergoing anoikis and makes tumors more aggressive [14, 15]. In EOC [16], cytoplasmic p120ctn regulates the activation of the Rho GTPases RhoA, Rac1 and Cdc42, which are known to be essential modulators for cell migration and invasion and consequently promoting cancer cell motility and invasion. In addition, the nuclear entry of p120ctn enables its binding with Kaiso, a transcriptional repressor, which has an important role in cell invasion and cancer aggressiveness [17]. The interaction between p120ctn and Kaiso is not only able to activate gene transcription of tumor suppressors but also to induce the pro-tumorigenic and pro-invasive canonical Wnt signaling pathway [18, 19]. However, the role of p120ctn in cancer needs to be further elucidated.

MUC1 has been shown to promote tumorigenesis. The MUC1 cytoplasmic domain can interact with β-catenin to modulate oncogenic signaling cascades [20]. MUC16 shares many structural similarities with MUC1; it has a SXXXXXSSX motif in its C-terminus similar to MUC1 which is crucial for the interaction between MUC1 and β-catenin [21]. A recent research also identifies the endogenous interaction between a C-terminal fragment of MUC16 and β-catenin in promoting tumorigenesis and metastasis [22]. Besides, it has also been found that the MUC16 CTD could increase the degradation of β-catenin by interacting with β-catenin to enhance the formation of multicellular aggregates, which promotes tumor progression in EOC [7]. Both β-catenin and p120ctn belong to the catenin family and have familiar structures. In pancreatic cancer, the carboxyl terminal cytoplasmic tail of MUC1 (MUC1 CT) is also demonstrated to bind to p120ctn to promote cell adhesion, motility and metastasis [23]. However, the relationship between MUC16 and p120ctn in EOC development is poorly understood.

In this study, we find that MUC16 and p120ctn are overexpressed in OC patients. The aberrantly expressed MUC16 CTD in EOC cells promotes proliferation and migration abilities by translocating p120ctn to the cytoplasm, activating RhoA/Cdc42. Meanwhile, the knockout (KO) of MUC16 inhibits this reaction. Our study is the first one reporting the interaction between MUC16 and p120ctn in the process of EOC development. These results may provide a basic mechanism for targeted therapy of EOC.

## Methods

### Cell lines and lentivirus transfection

The SKOV3 and HO8910 EOC cell lines were purchased from the Cell Bank of the Chinese Academy of Science (Shanghai, China). Cells were cultured in RPMI 1640 medium (Invitrogen, CA, USA) with 10% FBS (Invitrogen, CA, USA). The cells were maintained under a 5% CO_2_ atmosphere at 37 °C. For the overexpression of MUC16 CTD in SKOV3 cells, an N-terminal FLAG-tagged polypeptide consisting of the carboxyl-terminal 114 amino acids of MUC16 (MUC16 CTD) was stably overexpressed in SKOV3 cells through lentiviral transfection, and the empty vector (EV) was also transfected into cells as a negative control. DNA fragments encoding the carboxyl-terminal region of MUC16 (114 amino acids) were generated according to previous research [2]. Lentiviral vectors for the human MUC16 CTD expression sequence were constructed by Hanyin Co. (Shanghai, China). Lentiviral vectors for human p120ctn-shRNA carrying a green fluorescent protein (GFP) sequence were constructed by Hanyin Co. (Shanghai, China). The target sequence for human p120ctn knockdown was as follows: p120ctn-shRNA: 5′-TAGCTGACCTCCTGACTAA-3′. Then, the p120ctn-shRNA was transfected into SKOV3 MUC16 CTD cells and HO8910 negative control (NC) cells to KD the expression of p120ctn.

### CRISPR/Cas9 genome-editing system gene deletion

Lentiviral vectors expressing Cas9 and single guide RNAs (sgRNAs) targeting MUC16 were constructed by Hanyin Co. (Shanghai, China). The guide sequences used were 5′-GACATCTACAGGTGCAATCG-3′ for sgRNA1 and 5′-AGAGGGAGTTCCATTGACC-3′ for sgRNA2. Cells were then infected with the lentivirus. After 72 h, puromycin (2 μg/ml) was added for 3 days. After recovering for 4–6 days, individual colonies were picked and genotyped by DNA sequencing of the amplified KO region. Immunoblot analysis was also performed to confirm the lack of MUC16 protein.

### TMA and IHC

BOT tissues and OC specimens were obtained from Shanghai First Maternity and Infant Hospital, Tongji University (Shanghai, China). Approved by the Ethics Committee of Shanghai First Maternity and Infant Hospital, the TMA of BOT consists of 86 benign ovarian tumor specimens (47 serous cystadenoma and 39 mucinous cystadenoma), and the OC TMA consists of 116 samples of malignant ovarian tumor tissues (Additional file 1: Table S1). Informed consent was obtained. All the tissues collected during surgery were fixed in formalin and then made into paraffin blocks. A 2.0 mm core of each paraffin block was collected in the appropriate TMA. The completed TMAs were cut into 4 μm-thick sections and mounted on slides. For the immunohistochemistry (IHC), primary antibodies against MUC16 (1:100, Abcam, Cambridge, UK) and p120-catenin (1:500, Abcam, Cambridge, UK) were utilized, and goat anti-rabbit IgG-AP with Fast-Red (PicTureTMDouble Staining Kit, Invitrogen) was used as the secondary antibody. The staining intensity was rated on a scale of 0–3 in which 0 = none, 1 = +weak, 2 = ++ intermediate, and 3 = +++ strong. The proportion score was found by analyzing the percentage of positively stained cells: 0 for none, 1 for < 25%, 2 for 25–50%, 3 for 50–75%, and 4 for > 75%. Data were transformed into the German immunoreactive score (IRS) by multiplying the staining intensity scores and proportion scores. The final protein expression scores ranged from 0 to 12: 0–3 = weak, 4–6 = moderate, > 7 = strong. The immunohistochemical results were evaluated manually by two different pathologists.

### Western blotting and subcellular protein extraction

Equal amounts of proteins (20 μg) lysed from cells were resolved in SDS-PAGE gel and then transferred to nitrocellulose membranes (Millipore, Bedford, MA). Primary antibodies against MUC16 (1:10000, Abcam, Cambridge, UK), p120-catenin (1:1000, Abcam, Cambridge, UK), FLAG (1:500, Proteintech Group, Inc., IL, USA), GAPDH (1:1000, Cell Signaling Technology, Beverly, MA), β-actin (1:1000, Abcam, Cambridge, UK), lamin B1 (1:500, Abcam, Cambridge, UK), Rac1 (1:1000, Abcam, Cambridge, UK), Cdc42 (1:125, Abcam, Cambridge, UK), and RhoA (1:5000, Abcam, Cambridge, UK) were used. The synthetic peptide of primary antibodies against MUC16 corresponding to Human MUC16 aa 12,450–12,550. Anti-rabbit or anti-mouse IgG (1:2000, Cell Signaling Technology, Beverly, MA) was used as the secondary antibody. For subcellular fractions, cytoplasmic or nuclear proteins were extracted using a cytoplasmic protein extraction kit or a nuclear protein extraction kit (SAB, MD, USA). The purity of subcellular fractions was confirmed using antibodies against GAPDH (a cytoplasmic marker) and lamin B1 (a nuclear marker).

### Real-time quantitative PCR

Total cellular RNA was extracted using TRIzol (Invitrogen, CA, USA), and mRNAs were reverse transcribed into cDNA with a PrimeScript™ RT Reagent Kit (Perfect Real Time) (Takara, Japan). SYBR® Premix Ex Taq™ (TliRNaseH Plus) (Takara, Japan) was used to quantify cDNA, and gene expression levels were calculated by the ΔΔCt method. mRNA results were normalized to GAPDH. The primer sequences used are as follows: MUC16 CTD: forward: 5′-CCCTGAGAAATTTTGGAGTTTC-3′, reverse: 5′- GGACTGTGTACTTCTCAGTGACTG-3′; GAPDH: forward: 5′-CTGGGCTACACTGAGCACC-3′, reverse: 5′-AAGTGGTCGTTGAGGGCAATG-3′; p120-catenin: forward: 5′-GTGACAACACGGACAGTACAG-3′,reverse: 5′-TTCTTGCGGAAATCACGACCC-3′.

### Immunofluorescence

Cells were plated on glass slides and grown to 50–70% confluence. Then, the cells were washed 3 times with cold PBS and fixed in 4% formaldehyde for 15 min at room temperature. After the cells were permeabilized in PBS with 1% Triton X-100 and blocked with 5% bovine serum albumin for 1 h, the glass slides were incubated in antibodies against p120-catenin (1:100) or antibodies against FLAG (1:100) for 2 h at room temperature. The cells were washed 3 times with PBS containing 0.2% Triton X-100 and then followed by fluorescent-labeled secondary antibodies for 1 h. The nuclei were co-stained with 1 μg/ml DAPI. The final images were captured with a Nikon Eclipse E600 fluorescence microscope.

### MTS proliferation assay and cell migration assay

For the MTS proliferation assay, 2 × 10^3^ cells were seeded in a 96-well plate for 72 h and then incubated with 20 μl of MTS Solution Reagent (Promega Biosciences, CA, USA) for 2 h. The absorbance at 492 nm was recorded using a 96-well plate reader. To assess the ability of the cells to migrate, we seeded 2 × 10^4^ SKOV3 cells or 5 × 10^4^ HO8910 cells in 24-well transwell chambers (Corning Inc., USA) with 8-μm inserts. After the cells were incubated at 37 °C for 16 h, crystal violet 1% was used to stain the cells that had migrated through the membrane, and cells were counted in 4 random fields using ImageJ.

### Rho GTPase activity assay

The activation of Rho GTPases was analyzed using an Active Rho Detection Kit (Cell Signaling Technology, Beverly, MA). The GTP-bound GTPase pull-down process was performed according to the manufacturer’s instructions. The eluted sample was then analyzed by western blotting. Anti-Rac1, anti-Cdc42, anti-RhoA and anti-β-actin antibodies were used.

### Statistical analysis

All the data are presented as the mean ± SEM and shown as error bars. Student’s t-test or a chi-squared test was performed in SPSS 20.0 to compare the differences between groups. Only a significance value of *p* < 0.05 was regarded as statistically significant.

## Results

### Expressions of MUC16 and p120ctn in OC tissue specimens

**Fig. 1:**
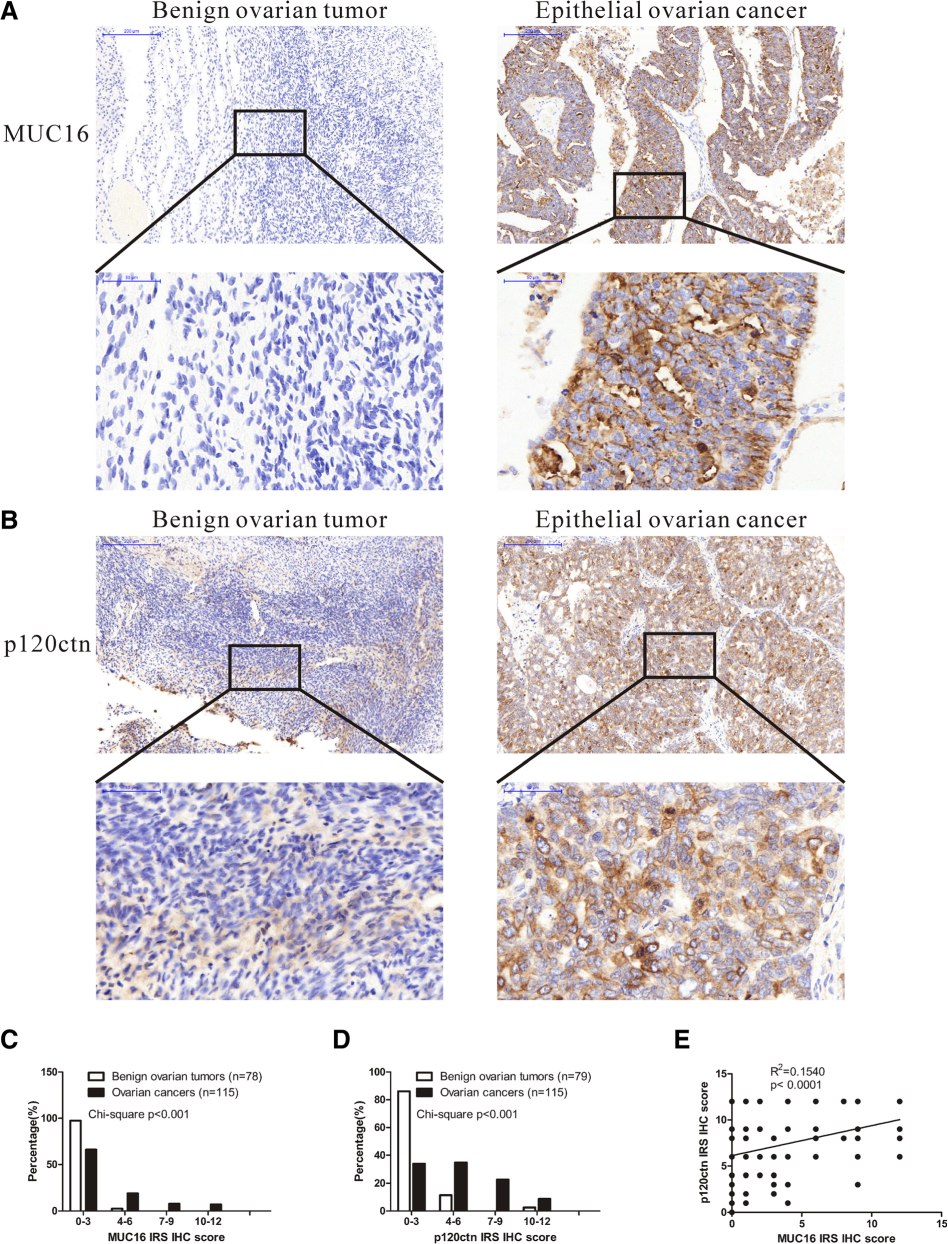
Both MUC16 and p120ctn are aberrantly overexpressed in OC tissue samples compared with BOT. **a** Representative IHC examples of MUC16 expression levels in BOT and OC specimens are shown. **b** Representative IHC examples of p120ctn expression levels in BOT and OC specimens are shown. **c**, **d** IRS classifications of MUC16 and p120ctn are shown. Data are shown as the mean ± SEM (*n* = 3). **e** Correlation between MUC16 and p120ctn in EOC samples is analyzed. R^2^ is calculated by linear regression

To evaluate the relationship among MUC16, p120ctn and OC in patients, we analyzed the expression levels of MUC16 and p120ctn in benign ovarian tumors (BOT) and OC tissue specimens. The tissue microarray (TMA) of BOT consists of 86 BOT specimens (47 serous cystadenoma and 39 mucinous cystadenoma), and the TMA of OC consists of 116 specimens. The characteristics of OC TMA are shown in Additional file 1: Table S1, and the correlation between clinic pathological characteristics and MUC16 or p120ctn expression is analyzed in Additional file 1: Table S2. Immunohistochemical analysis of TMAs showed that, compared with BOT, OC tissue specimens have stronger immunoreactivity of both MUC16 and p120ctn. These results reveal that OC patients express higher levels of MUC16 and p120ctn than BOT patients (Fig. 1a-d). We also analyze the linear relation between MUC16 expression level and p120ctn expression level (Fig. 1e).

### Overexpression of the MUC16 CTD promotes, and the deletion of MUC16 inhibits, the proliferation and migration abilities of EOC cells

**Fig. 2:**
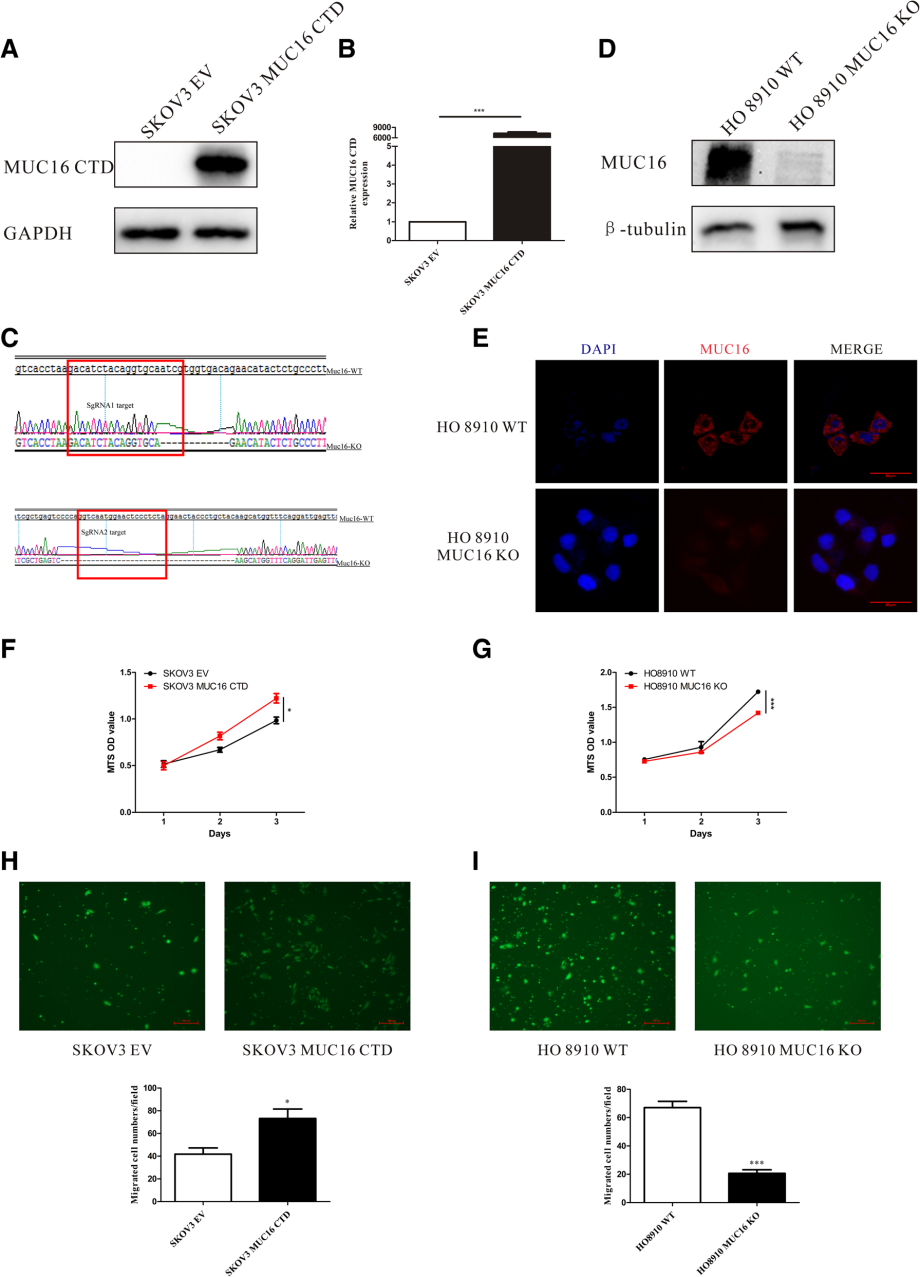
MUC16 CTD promotes, while the deletion of MUC16 inhibits, the proliferation and migration of EOC cells. SKOV3 cells were stably transfected with MUC16 CTD overexpression lentiviral vectors or empty vectors (EV). **a** Western blot is used to evaluate the expression of MUC16 CTD. **b** Real-time PCR is performed to analyze the RNA level of MUC16 CTD. Data are shown as the mean ± SEM (n = 3). ***p < 0.001 compared with the negative control (SKOV3 EV). **c** To knock out MUC16 in HO8910 cells, we designed two single guide RNA (sgRNA) targets to ensure the effect of MUC16 KO. **d** Knockout of MUC16 is also confirmed by western blot. **e** the downregulation of MUC16 in HO8910 cells is also confirmed by immunofluorescence. Data shown here are representative of 3 independent experiments. **f**, **g** The proliferation capacities of SKOV3 MUC16 CTD and HO8910 MUC16 KO are analyzed using MTS proliferation assay. Cell viability is determined by OD value. Data are shown as the mean ± SEM (*n* = 3). *p < 0.05 compared with SKOV3 EV. ***p < 0.001 compared with HO8910 WT. **h**, **i** A transwell assay is used to analyze the migration capacity of SKOV3 MUC16 CTD or HO8910 MUC16 KO cells; migrated cells are counted. Data are shown as the mean ± SEM (*n* = 3). *p < 0.05, ***p < 0.001. Data shown here are representative of 3 independent experiments

Previous studies have found that the overexpression of MUC16 promotes EOC development and that the MUC16 CTD plays an important role in the process [5–7, 22, 24, 25]. To analyze the relationship between MUC16 and p120ctn during EOC development, we overexpressed the MUC16 CTD in SKOV3 cells which were well known for lack of MUC16 expression [7], and generated MUC16 KO HO8910 cells using CRISPR (clustered regularly interspaced short palindromic repeats) /Cas9 genome-editing technology [26]. After lentivirus transfection, the FLAG-tagged MUC16 CTD was highly and stably expressed in SKOV3 cells at both the protein level and the mRNA level (Fig. 2a, b). Due to the high-molecular-weight, all previous studies of MUC16 only used knockdown (KD) technology to study MUC16 deficiency in cancer. To our knowledge, this study is the first one using CRISPR/Cas9 genome-editing technology to KO MUC16 in EOC cells. We designed two single guide RNA (sgRNA) targets to ensure the effectiveness of MUC16 KO (Fig. 2c). Both two sgRNAs were infected into cells and successful KO was also verified by western blot (Fig. 2d). The downregulation of MUC16 in HO8910 cells was also further confirmed by immunofluorescence (Fig. 2e). This type of deletion of MUC16 might help us to analyze the biological function of MUC16 more comprehensively with respect to cancer, especially in EOC. Then, to analyze the relationship between MUC16 and EOC, we studied the proliferation abilities of MUC16 CTD overexpressing SKOV3 cells (SKOV3 MUC16 CTD) and MUC16 deleted HO8910 cells (HO8910 MUC16 KO). We found that the overexpressing of MUC16 CTD promoted SKOV3 cells proliferation (Fig. 2f) and the deletion of MUC16, conversely, inhibited the proliferation ability of HO8910 cells (Fig. 2g). Additionally, we analyzed the migration ability of the cells; consistent with proliferation results, overexpression of MUC16 CTD enhanced, while deletion of MUC16 inhibited, EOC cell migration (Fig. 2h, i).

### The subcellular localization of p120ctn is regulated by MUC16 in EOC cells

**Fig. 3:**
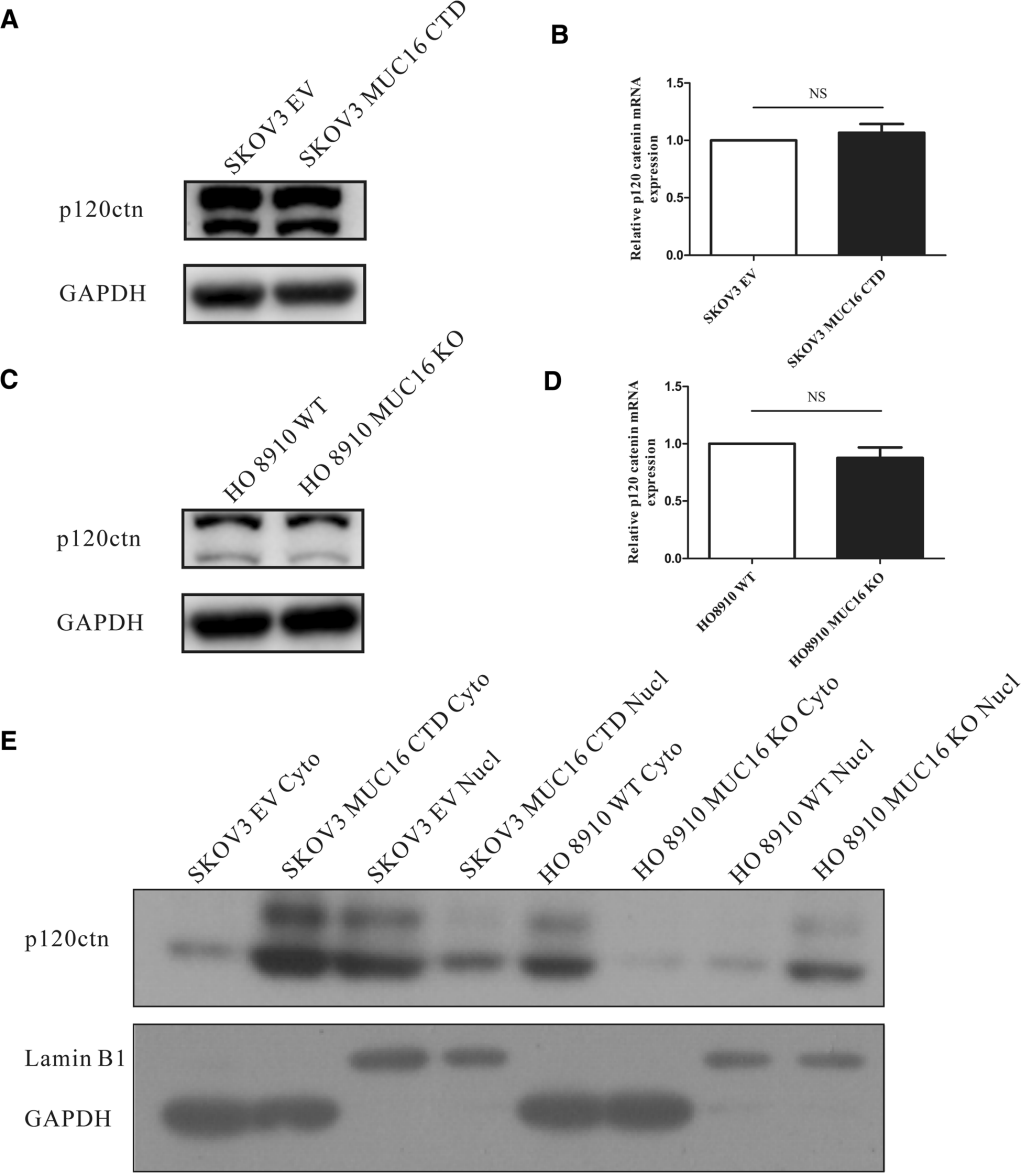
MUC16 promotes cellular cytoplasmic accumulation of p120ctn without influencing the total expression level of p120ctn in EOC cells. **a** The total cellular p120ctn protein levels of SKOV3 MUC16 CTD cells are analyzed by western blot. Both bands are p120ctn**. b** The total RNA levels are confirmed by real-time PCR. Data are shown as the mean ± SEM (*n* = 3). Nonsignificant (NS). **c** The total cellular p120ctn protein levels of HO8910 MUC16 KO cells are analyzed by western blot. **d** The total RNA levels are confirmed by real-time PCR. Data are shown as the mean ± SEM (*n* = 3). Nonsignificant (NS). Data shown here are representative of 3 independent experiments. **e** Cytoplasmic or nuclear proteins are extracted and analyzed by western blot

P120ctn has been shown to be associated with cell adhesion, motility, invasion and proliferation in cancer [10, 12, 16, 27]. The tumor-promoting function of p120ctn is regulated by MUC1 [23], another well-known member of the MUC family, which is structurally similar to MUC16. Additionally, we find that EOC patients have higher expression level of p120ctn, consistent with MUC16 (Fig. 1). Therefore, we speculate that p120ctn may play an important role in the proliferation and migration of EOC cells induced by MUC16. To evaluate the potential interaction between MUC16 with p120ctn, we tested the protein and mRNA levels of p120ctn in SKOV3 MUC16 CTD cells and HO8910 MUC16 KO cells. We found that the total protein and mRNA expression levels of p120ctn remained unchanged both in SKOV3 MUC16 CTD cells (Fig. 3a, b) and HO8910 MUC16 KO cells (Fig. 3c, d). Then, to analyze whether the subcellular localization of p120ctn could be regulated by MUC16, we isolated the cytoplasmic and nuclear proteins of cells using a cytoplasmic/nuclear protein extraction kit. In Fig. 3e, we showed that overexpression of the MUC16 CTD increased the level of p120ctn in the cytoplasm but decreased the nuclear expression level of p120ctn. Moreover, the deletion of MUC16 abolished the cytoplasmic accumulation of p120ctn (Fig. 3e). These results indicate that MUC16 contributes to the cytoplasmic translocation of p120ctn in EOC cells.

### The cell proliferation and migration induced by MUC16 are mediated by p120ctn through RhoA/Cdc42 activation

**Fig. 4:**
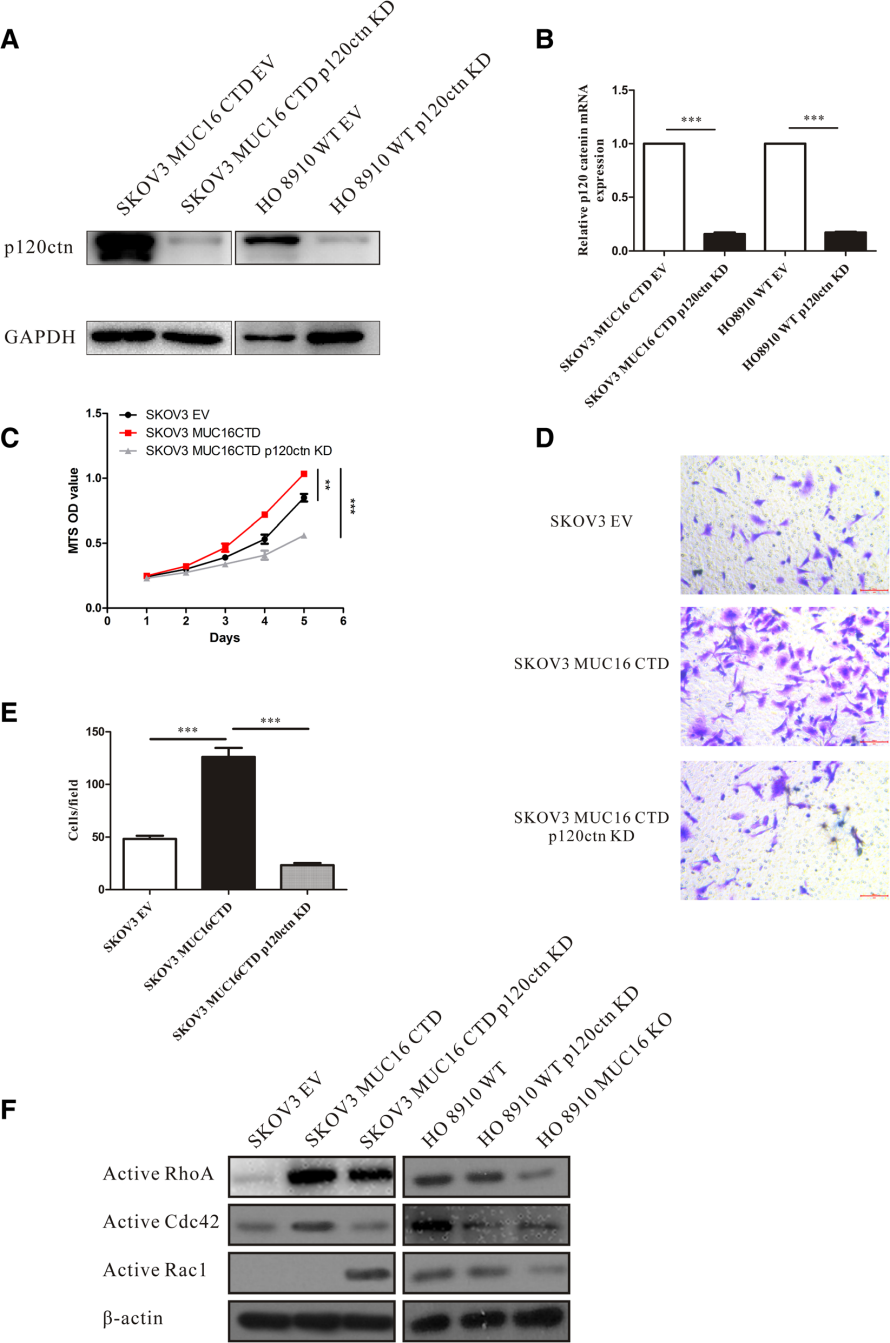
The cell proliferation and migration induced by MUC16 is mediated by p120ctn through RhoA and Cdc42 activation. Cells expressing high levels of MUC16 CTD or MUC16 were stably transfected with p120ctn-shRNA-lentivirus vectors or EV. **a** Western blot is used to analyze the proteins. **b** Real-time PCR is used to analyze the RNA expression level of p120ctn. Data are shown as the mean ± SEM (*n* = 3). ***p < 0.001. **c** The proliferation capacities of SKOV3 EV, SKOV3 MUC16 CTD and SKOV3 MUC16 CTD p120ctn KD are analyzed using MTS proliferation assay. Cell viability is determined by OD value. Data are shown as the mean ± SEM (*n* = 3). **p < 0.01. ***p < 0.001. **d**, **e** The migration capacity of SKOV3 EV, SKOV3 MUC16 CTD and SKOV3 MUC16 CTD p120ctn KD are also shown. ***p < 0.001. **f** Cells are subjected to pull-down assays. GTP-bound (active) RhoA, Cdc42, and Rac1 are analyzed by western blot. Data shown here are representative of 3 independent experiments

Given that cytoplasmic p120ctn can regulate cell proliferation and migration through activatiing Rho family GTPases [28, 29], to further verify the promoted proliferation and migration abilities of EOC cells induced by MUC16 CTD are mediated by the cytoplasmic p120ctn, we analyzed the relationships among MUC16, p120ctn and Rho GTPases. Lentiviral vectors carrying human p120ctn-shRNA were transfected into cells to KD p120ctn (Fig. 4a, b). We analyzed the proliferation and migration abilities of p120 ctn KD cells. The results showed that the lack of p120 ctn inhibited the promotion function induced by MUC16 CTD (Fig. 4c, d, e). Then, we utilized an Active Rho Detection Kit to test the activity of Rho GTPases (RhoA, Cdc42, Rac1) in SKOV3, SKOV3 MUC16 CTD, SKOV3 MUC16 CTD p120ctn KD, HO8910, HO8910 p120ctn KD and HO8910 MUC16 KO cells. The western blot results are shown in Fig. 4f. As shown in Fig. 4f, the over expression of MUC16 CTD in SKOV3 promotes the active RhoA and active Cdc42 expressions while the KD of p120ctn inhibits this function. In HO8910 cells, compared with HO8910 WT, either the p120ctn KD or the KO of MUC16 decreased the active RhoA and active Cdc42 expressions.

## Discussion

It is believed (but not proven) that MUC16 undergoes cleavage in the penultimate SEA domain to generate circulating CA125 [24], which is well known for its application as a clinical biomarker for the diagnosis of tumor recurrence, especially for EOC. Due to the large mass of MUC16, only a limited amount has been revealed about its mechanism and function in cancer. In the present study on the detailed functional role of MUC16 in EOC, we find that MUC16 and p120ctn are aberrantly expressed in 94 clinical OC samples compared with BOT (Fig. 1). OC patients express higher levels of MUC16 and p120ctn than BOT patients, which implies aberrant overexpressed MUC16 and p120ctn may play an important role in OC development. We also provide evidence that MUC16 is a critical inducer of the proliferation and migration of EOC cells and that the CTD of MUC16 plays an important role in these processes (Fig. 2). To the best of our knowledge, this is the first study that uses the CRISPR/Cas9 genome-editing system to study the biological function of MUC16 in EOC. In addition, we reveal the previously unstudied relationship between MUC16 and p120ctn in EOC. We showed that MUC16 promoted the translocation of p120ctn to the cytoplasm, where it activated Rho GTPases to modulate the proliferation and migration abilities of EOC cells (Figs. 3, 4).

MUC16 is found to be overexpressed in various cancers including pancreatic carcinoma, breast cancer, non-small-cell lung cancer, and especially EOC [30]. Initial research showed that MUC16 interacted with mesothelin on the surface of mesothelial cells to enhance peritoneal metastasis in EOC [31]. In addition, MUC16 participated in cancer cell immune evasion by interacting with natural killer cells, B cells, and monocytes via Siglec-9 [32]. Another study also found that the loss of MUC16 stimulated the EMT of EOC cells [25]. However, the specific mechanism still needs to be elucidated. To further analyze the function of MUC16 in the development of EOC, we collected 94 samples of OC tissues and used them to construct a TMA to test the expression level of MUC16 in OC (Fig. 1). We found that OC patients had much higher MUC16 expression levels than patients with BOT (serous or mucous cystadenoma). In this study, we also showed that overexpression of MUC16 CTD enhanced the proliferation and migration abilities of SKOV3 cells, while the deletion of MUC16 reversed this effect. These results highlight the tumor-promoting function of MUC16 in EOC (Fig. 2). These results imply that the aberrant expression of MUC16 plays an important role in the development of EOC.

First discovered as a substrate of the Src oncogene, p120ctn, which, along with β-catenin, is a member of the armadillo (ARM) protein family, is well known for binding and stabilizing cadherins [33]. P120ctn is a critical regulator of adherens junctions (AJs) between cells, as well as cell morphogenesis and immunity in mammals [34]. In cancer, the downregulation or subcellular translocation of p120ctn is closely related to tumorigenesis [35]. Cytoplasmic p120ctn was found to interact with Rho GTPases RhoA, Rac1 and Cdc42 to influence cancer cell motility and metastasis [15]. In addition, p120ctn within the nucleus was able to suppress Kaiso, a transcription repressor that can inhibited the Wnt signaling pathway [33]. However, the expression level of p120ctn in EOC patients has not been studied. Thus, we also investigated the expression levels of p120ctn in BOT and OC TMAs (Fig. 1b, d). Interestingly, similar to MUC16, p120ctn was significantly more highly expressed in OC than in BOT. P120ctn may also play an important role in OC tumorigenesis. We also analyzed the linear relation between MUC16 expression level and p120ctn expression level (Fig. 1e). The result showed there was no specific linear relation between MUC16 and p120ctn, which implied that MUC16 may modulate p120ctn indirectly in EOC.

To further analyze the potential mechanism of overexpressed MUC16 and p120ctn in EOC tumorigenesis, we referred to previous studies and found that MUC16 regulated the translocation of β-catenin, which activated the Wnt signaling pathway, protecting EOC cells from anoikis when they were separated from their matrix [7]. P120ctn and β-catenin belong to the same catenin family and have a high degree of structural similarity. Apart from these, another MUC member, MUC1, which has a similar structure to MUC16, interacts with p120ctn to regulate the dynamic features of cell adhesion, motility and metastasis in pancreatic cancer [23]. Previous findings have revealed the critical impact of p120ctn in cell proliferation [36] and motility [37] via Rho GTPase activity. However, the interaction between MUC16 and p120ctn has not yet been reported. We hypothesize that the facilitating role of MUC16 in EOC cell proliferation and migration is mediated by p120ctn.

Then, to verify this hypothesis, we analyzed the total expression level of p120ctn in SKOV3 MUC16 CTD and HO8910 MUC16 KO cells. Interestingly, although the expression levels of MUC16 and p120ctn were consistent among EOC tissue samples, total p120ctn expression in EOC cells in vitro is not influenced by the expression of MUC16 (Fig. 3a-d). These results are consistent with Fig. 4e, which suggest that there may be an indirect interaction between MUC16 and p120ctn in EOC. Studies on p120ctn in most human cancers have found that not only its expression level but also its subcellular translocation led to its pro-tumorigenic effect [12]. Additionally, a recent study showed that gonadotropin-releasing hormone regulates p120ctn cytoplasmic translocation to promote cancer cell migration and invasion via Rac1 and Cdc42 [16]. Thus, on the basis of the unchanged total level of p120ctn, we suggested that MUC16 might impact the translocation of p120ctn in EOC cells. We isolated the cytoplasmic and nuclear proteins of cells and found that p120ctn was recruited to the cytoplasm by MUC16 CTD and the loss of MUC16 reversed this effect (Fig. 3e). The expression of p120ctn in cytoplasm of SKOV3 MUC16 CTD or HO8910 WT cells was higher than SKOV3 EV or HO8910 MUC16 KO cells. And this phenomenon was precisely opposite in nuclear fraction of SKOV3 MUC16 CTD or HO8910 WT cells. These results imply that overexpressed MUC16 in EOC plays an important role in promoting cytoplasmic aggregation of p120ctn without influencing total protein expression level of p120ctn. Our findings highlight the existence of a potential interaction between MUC16 and p120ctn cellular translocation. However, the detailed mechanism still needs to be further analyzed. To our knowledge, our study is the first one reporting the relationship between MUC16 and p120ctn cellular translocation in EOC.

Finally, to further analyze the relationship between cytoplasmic p120ctn and enhanced proliferation and migration abilities induced by MUC16, we knocked down p120ctn expression in SKOV3 MUC16 CTD and HO8910 WT cells. Results showed that the promoting effects of MUC16 were significantly inhibited in p120ctn KD cells (Fig. 4c, d, e). These results demonstrated that p120ctn was a crucial mediator of MUC16 induced tumorigenesis. Above results have showed (Figs. 2, 3), MUC16 was able to increase cytoplasmic aggregation of p120ctn indirectly without influencing total protein expression level of p120ctn in EOC cells. In order to further study the mechanism of cytoplasmic p120ctn in modulating MUC16 promoted proliferation and migration, we referred to a lot of related researches and found that the cytoplasmic p120ctn could affect cancer development through modulating Rho GTPases activation [11, 12, 28, 29, 37]. Plenty of researches have proved the crucial functions of Rho GTPases activation in OC development [16, 38–40]. The tumorigenesis and progression of ovarian carcinoma were regulated through RhoA Pathway, the inhibition of RhoA inhibited ovarian cancer cell proliferation, metastasis and invasion [38]. Besides, Cdc42 knockdown significantly reduced cell proliferation and notably inhibited the adhesion, motility and invasiveness in ovarian cancer [39]. As for ovarian cancer chemotaxis, RhoA and Rac1 were suggested to play independent roles in the chemotactic migration of ovarian cancer cells, inhibition of RhoA and Rac1 decreased cancer cell migration in LPA-induced chemotaxis [40]. We tested the activation of Rho GTPases in EOC cells. The results showed that the overexpression of MUC16 CTD promoted RhoA/Cdc42 activation while the KD of p120ctn inhibited the activation. In addition, the deletion of MUC16 in HO8910 WT significantly reduced RhoA/Cdc42 activation (Fig. 4f). Therefore, these findings suggest that the EOC cell proliferation and migration induced by MUC16 is mediated by the cytoplasmic translocation of p120ctn via RhoA/Cdc42 activation.

## Conclusions

In summary, we tested the expression level of MUC16/p120ctn in OC patient samples and revealed the novel role of MUC16 in regulating the proliferation and migration of cells during EOC development. Our data also suggest a potential relationship between MUC16 and p120ctn. To the best of our knowledge, this is the first report showing how the subcellular translocation of p120ctn is impacted by MUC16 in cancer. The promoting function of MUC16 in the proliferation and migration of EOC cells is mediated by cytoplasmic translocated p120ctn through RhoA/Cdc42 activation. These results may provide new insights into mechanisms related to EOC development. This knowledge may also be useful for exploiting new therapeutic targets for EOC.

## Additional file

Additional file 1: **Table S1.** Characteristics of tumor tissue from patients diagnosed with ovarian cancer. **Table S2.** Association of MUC16 and p120ctn expression with clinicopathological characteristics of ovarian cancer. (DOC 45 kb)

## Abbreviations

BOT: Benign ovarian tumor
CTD: Cytoplasmic tail domain
EMT: Epithelial mesenchymal transition
EOC: Epithelial ovarian cancer
EV: Empty vector
IHC: Immunohistochemistry
KD: Knock down
KO: Knock out
p120ctn: p120-catenin
Rho: Ras homologus oncogene
SEA: Sea urchin sperm, enterokinase, and agrin
TMA: Tissue microarray
