# Supplementary material for: MUC16 impacts tumor proliferation and migration through cytoplasmic translocation of P120-catenin in epithelial ovarian cancer cells: an original research
Source: BMC Cancer. 2019 Feb 22;19:171. doi: 10.1186/s12885-019-5371-4 (PMC6387523; doi:10.1186/s12885-019-5371-4)
Supplement: Supplementary file 1 — Table S1. Characteristics of tumor tissue from patients diagnosed with ovarian cancer. Table S2. Association of MUC16 and p120ctn expression with clinicopathological characteristics of ovarian cancer. (DOC 45 kb) [file 12885_2019_5371_MOESM1_ESM.doc]

**Supplementary Table 1**: Characteristics of tumor tissue from patients diagnosed with ovarian cancer

| Characteristics | Ovarian cancer (N=116) |
| --- | --- |
|  | N (%) |
| *Age*  <50  ≥50  No information  *FIGO stage*  I  II  III  IV  No information  *Histological grade of tumor*  Low/moderate  High  No information  *Tumor cell type*  Serous adenocarcinoma  Borderline adenocarcinoma  Clear-cell carcinoma  Non-epithelial ovarian cancers | 41(35)  68(59)  7(6)  35(30)  14(12)  38(33)  3(3)  26(22)  40(34)  45(39)  57(49)  16(14)  21(18)  21(18) |

**Supplementary Table 2**: Association of MUC16 and p120ctn expression with clinicopathological characteristics of ovarian cancer

| Characteristics | MUC16 | | | P120ctn | | |
| --- | --- | --- | --- | --- | --- | --- |
|  | Low expression(n) | High expression(n) | *p value* | Low expression(n) | High expression(n) | *p value* |
| *Age* |  |  | 0.637 |  |  | <0.05 |
| <50 | 36 | 5 |  | 36 | 5 |  |
| ≥50 | 66 | 12 |  | 52 | 26 |  |
| *Ascites* |  |  | - |  |  | 1.000 |
| Yes | 1 | 0 |  | 1 | 0 |  |
| No | 22 | 0 |  | 18 | 3 |  |
| *FIGO stage* |  |  | 0.072 |  |  | 0.569 |
| I-II | 44 | 5 |  | 35 | 14 |  |
| III-IV | 31 | 10 |  | 27 | 14 |  |
| *Histological grade of tumor* |  |  |  |  |  |  |
| Low/moderate | 33 | 7 | 0.809 | 31 | 9 | 0.414 |
| High | 38 | 7 |  | 38 | 7 |  |
| *Serum CA125 (median, range)* | 100.95, 8.60-1000 | 113.20, 35.10-191.30 | 0.405 | 180.40, 22.40-1000 | 48.57, 8.60-92.90 | 0.135 |
| *Tumor cell type* |  |  | 0.591 |  |  | <0.05 |
| Serous adenocarcinoma | 46 | 11 |  | 41 | 16 |  |
| Borderline adenocarcinoma | 14 | 2 |  | 16 | 0 |  |
| Clear-cell carcinoma | 19 | 2 |  | 14 | 7 |  |
| Non-epithelial ovarian cancers | 19 | 2 |  | 10 | 11 |  |
